# Cryptic Diversity of the European Blind Mole Rat *Nannospalax leucodon* Species Complex: Implications for Conservation

**DOI:** 10.3390/ani12091097

**Published:** 2022-04-23

**Authors:** Vanja Bugarski-Stanojević, Gorana Stamenković, Vida Jojić, Nada Ćosić, Duško Ćirović, Oliver Stojković, Jelena Veličković, Ivo Savić

**Affiliations:** 1 Department of Genetic Research, Institute for Biological Research “Siniša Stanković”—National Institute of Republic of Serbia, University of Belgrade, 11060 Belgrade, Serbia; gorana.stamenkovic@ibiss.bg.ac.rs (G.S.); vjojic@ibiss.bg.ac.rs (V.J.); 2 Department of Evolutionary Biology, Institute for Biological Research “Siniša Stanković”—National Institute of Republic of Serbia, University of Belgrade, 11060 Belgrade, Serbia; nada.cosic@ibiss.bg.ac.rs; 3 Institute of Zoology, Faculty of Biology, University of Belgrade, 11060 Belgrade, Serbia; dcirovic@bio.bg.ac.rs (D.Ć.); ir_savic@yahoo.com (I.S.); 4 Institute of Forensic Medicine, Faculty of Medicine, University of Belgrade, 11060 Belgrade, Serbia; oliver.stojkovic@med.bg.ac.rs; 5 Laboratory Diagnostics Citilab, 11060 Belgrade, Serbia; jelena.velickovic@citilab.rs

**Keywords:** chromosomal speciation, cryptic species, *16S rRNA* gene, *MT-CYTB* gene, evolutionary rates

## Abstract

**Simple Summary:**

Cryptic species, hidden by morphological uniformity, represent a significant part of the diversity in some taxonomic groups, and pose a real challenge for conservation planning. Here, we explore cryptic speciation in blind mole rats of the genus *Nannospalax*—comprehensively studied mammals for many unusual features (cancer resistance, longevity, etc.). Intensive chromosomal changes are one of these peculiarities. In the European *N. leucodon* species complex, 25 lineages with different karyotypes have been described, comprising undetected/cryptic species. As some of them are endangered, taxonomic revision is urgent for conservation purposes. Using 36–60-year-old archived teeth samples and newly captured animals, we analysed the nucleotide polymorphism of two mitochondrial gene sequences among 17 out of 25 chromosomal forms—the highest number studied so far—and provided molecular genetic records for 5 of them for the first time. Eleven chromosomal forms were separated into distinct clades in phylogenetic trees. High evolutionary divergence values among several chromosomal forms overlapped with those acquired for higher taxonomic categories. By integrating the results of previous karyological analyses and crossbreeding experiments that revealed complete reproductive isolation of seven chromosomal forms, with our new findings, we propose conservation strategies to preserve their genetic diversity.

**Abstract:**

We explored the cryptic speciation of the *Nannospalax leucodon* species complex, characterised by intense karyotype evolution and reduced phenotypic variability that has produced different lineages, out of which 25 are described as chromosomal forms (CFs), so many cryptic species remain unnoticed. Although some of them should be classified as threatened, they lack the official nomenclature necessary to be involved in conservation strategies. Reproductive isolation between seven CFs has previously been demonstrated. To investigate the amount and dynamics of genetic discrepancy that follows chromosomal changes, infer speciation levels, and obtain phylogenetic patterns, we analysed mitochondrial *16S rRNA* and *MT-CYTB* nucleotide polymorphism among 17 CFs—the highest number studied so far. Phylogenetic trees delineated 11 CFs as separate clades. Evolutionary divergence values overlapped with acknowledged higher taxonomic categories, or sometimes exceeded them. The fact that CFs with higher 2n are evolutionary older corresponds to the fusion hypothesis of *Nannospalax* karyotype evolution. To participate in conservation strategies, *N. leucodon* classification should follow the biological species concept, and proposed cryptic species should be formally named, despite a lack of classical morphometric discrepancy. We draw attention towards the *syrmiensis* and *montanosyrmiensis* CFs, estimated to be endangered/critically endangered, and emphasise the need for detailed monitoring and population survey for other cryptic species.

## 1. Introduction

Cryptic lineages are genetically distinct groups that show reduced morphological divergence. They represent a substantial part of the diversity in some geographic regions (e.g., tropical rainforests and marine habitats) and taxonomic groups (frogs, arthropods, etc.), and new ones are continuously being discovered [1]. With an expansion of broader geographic sampling as well as molecular techniques, primarily DNA barcoding—a tool for identifying and separating morphologically similar species—the capacity of scientists to describe and define biological diversity has changed, sometimes leading in another direction: “taxonomic over-inflation” [2,3]. However, speciation is not always followed by morphological changes, and various speciation mechanisms can initiate the diversification of new species through morphologically static cladogenesis [1]. Delimiting cryptic diversity is challenging, and influences evolutionary biology, biogeography, and conservation planning [4], so a thorough taxonomic knowledge based on the evolutionary history of a group is key to its conservation [3].

### 1.1. The Genus Nannospalax

Here, we explore cryptic speciation in the blind mole rat (BMR) genus *Nannospalax* (Palmer 1903) from the subfamily Spalacinae, whose taxonomy follows two different concepts: an older monogeneric model, and a two-generic model of the large and small-bodied BMRs—i.e., the greater BMR genus *Spalax* (Guldenstaedt, 1770), and the lesser BMR genus *Nannospalax*—that separates these two lineages according to morphological and karyological characteristics, as well as recent molecular and paleontological evidence, all reviewed in [5]. These subterranean mammals have developed many specific morphological and physiological adaptations due to surviving in an inhospitable environment. BMRs are model organisms for various research fields, e.g., tolerance to hypercapnia and hypoxia [6,7,8], cancer resistance and longevity [9,10], circadian rhythms [11,12], and sensory research [13]. Their specific lifestyle in cylindrical underground tunnels has equalised their morphological characteristics and limited their phenotypic differentiation. It was proposed that extreme environmental conditions may influence stabilising selection on morphology, reducing or eliminating morphological changes that usually follow speciation [1]. In addition, convergent morphology in the genus *Nannospalax* is combined with intensive chromosomal rearrangements (CRs) with diploid chromosomal numbers (2n) from 36 to 62. However, within a certain 2n value, chromosomal morphology between different population groups varies widely, causing different values (between 74 and 96) of the fundamental number of chromosomal arms (NF), i.e., the number of visible major chromosomal arms per set of chromosomes. According to 2n/NF differences, 74 basic chromosomal forms (CFs) have been named and described (reviewed in [14,15,16]). It was suggested that they most probably diverged through the process of allopatric speciation from a common ancestor, which possibly had 2n~60 and a high number of acrocentric chromosomes [16,17]. As speciation is a continuous process, CFs are detected in different evolutionary stages, occupying diverse distributional areas. Although some of them should be classified as threatened, they lack the official nomenclature necessary to be involved in conservation strategies and faunal listings, so the whole genus requires a taxonomic revision [18]. Moreover, solitary and territorial behaviour, low dispersal rates, and habitat fragmentation—mostly due to anthropogenic influence—deepen the genetic isolation of *Nannospalax* populations [19,20,21,22]. Still, their status on the IUCN Red List of Threatened Species stagnates at Data Deficient (DD) [23], acknowledging only three morphospecies/superspecies: (1) the European lesser BMR *Nannospalax leucodon* (Nordmann 1840), which inhabits parts of Central and Southeast Europe; (2) the Anatolian BMR *Nannospalax xanthodon* (Nordmann 1845) (synonym: *N. nehringi* (Satunin 1898) from Transcaucasia, Turkish Anatolia, and certain East Aegean islands; and (3) the Palestine BMR *Nannospalax ehrenbergi* (Nehring 1898), which occupies Southeastern Anatolia in Turkey, Iraq, Syria, Lebanon, Israel, Jordan, and Egypt. Within each of these superspecies, >20 CFs can be detected [14]. In addition to the three superspecies, the only CFs approved as separate species are *Spalax* (= *Nannospalax*) *galili*, *Spalax golani*, *Spalax carmeli*, and *Spalax judaei* from the *N. ehrenbergi* superspecies [24,25,26].

### 1.2. The Role of Chromosomal Changes in Reproductive Isolation

Intensive chromosomal rearrangements are common among rodents, particularly in the families Muridae and Cricetidae [27]. The highest numbers of CFs—72 and 97—were reported in the common shrew *Sorex araneus* and the house mouse *Mus musculus*, respectively [28,29]. However, karyotypic diversity in these two species is not reflected in molecular diversification due to its recent occurrence [30]. Despite reported genetic differentiation that follows karyotypic changes, two CFs of the common vole *Microtus arvalis* (*arvalis* and *obscurus*) are labelled as subspecies because they are not reproductively isolated [31]. Chromosomal rearrangements may act as barriers in the post-zygotic stage, and can be an important cause of cryptic speciation [32,33]. Pre- and post-copulation reproductive isolation between seven CFs of the European BMR *N. leucodon* (*serbicus*, *montanoserbicus*, *syrmiensis*, *montanosyrmiensis*, *hungaricus*, *monticola*, and *makedonicus*) was demonstrated by comprehensive experimental crossbreeding experiments, and additionally confirmed via artificial insemination [16,34]. Experimental crossbreeding results are also available in [5]. Only when the male and female with identical 2n/NF were combined (from the same and/or different populations) did copulation result in embryo development. Chromosome preparations made from the fibroblast culture showed no karyotypic differences between embryos and their parents. On the contrary, combinations of different CFs each time led to complete reproductive isolation, even in the case with identical 2n but different NF. To investigate the amount and dynamics of genetic discrepancy that follows chromosomal changes in this superspecies, detailed molecular studies are required.

### 1.3. Molecular Analysis of N. leucodon Genetic Differentiation

Compared to the other two *Nannospalax* superspecies (*N. xanthodon* and *N. ehrenbergi*), the European BMRs are the least explored. Recent molecular phylogenetic investigations that compared nucleotide sequence polymorphism of *16S rRNA* among eight *N. leucodon* CFs [35], along with *MT-CYTB* with two nuclear genes among six CFs [36], reported deep genetic divergence between some lineages, suggesting cryptic speciation among *N. leucodon* CFs. Besides, other studies analysed seven CFs and recorded lower genetic discrepancies among *N. leucodon* lineages compared to the two other superspecies [37,38]. Therefore, there is a real requirement for a more inclusive molecular investigation of this species complex.

This study investigates the taxonomic consequences of intensive chromosomal rearrangements by inferring genetic divergences and speciation levels among 17 *N. leucodon* CFs, embracing the highest number of European lesser BMR lineages studied to date. Molecular sequence data for five CFs (*strumiciensis* and *ovchepolensis* from North Macedonia, *epiroticus* and *hellenicus*, from Greece and *thracius* from Bulgaria) are presented here for the first time. This was accomplished by joining DNA extracted from fresh tissue, collected from newly sampled animals, and the teeth of old archived skulls. In order to increase the sequencing success rate from archived material, we used mtDNA genes. To obtain more realistic phylogenetic patterns, we analysed the amount of nucleotide polymorphism in two mitochondrial gene sequences—*16S rRNA* and *MT-CYTB*, which are functionally different and could have experienced differential mutational rates and selective constraints during evolution (for example, in Artiodactyla [39]). *16S rRNA* gene sequences have moderately well-conserved secondary structures among distantly related taxa [40], and are usually applied for distinguishing between well-resolved species and establishing relations between genera [41]. However, this gene was successfully used for differentiating cattle breeds [42]. Likewise, our previous investigation demonstrated that this approach is highly informative in the delineation of *Nannospalax* CFs [35]. Nevertheless, only one other study has explored *16S rRNA* gene polymorphism in this genus [38]. To expand the sample numbers in our sequence alignments and strengthen the inferred phylogenetic relations, we also analysed *MT-CYTB* gene sequences, since they have been broadly applied in this genus [14,36,37,43,44].

## 2. Materials and Methods

### 2.1. Sample Collection

**Figure 1:**
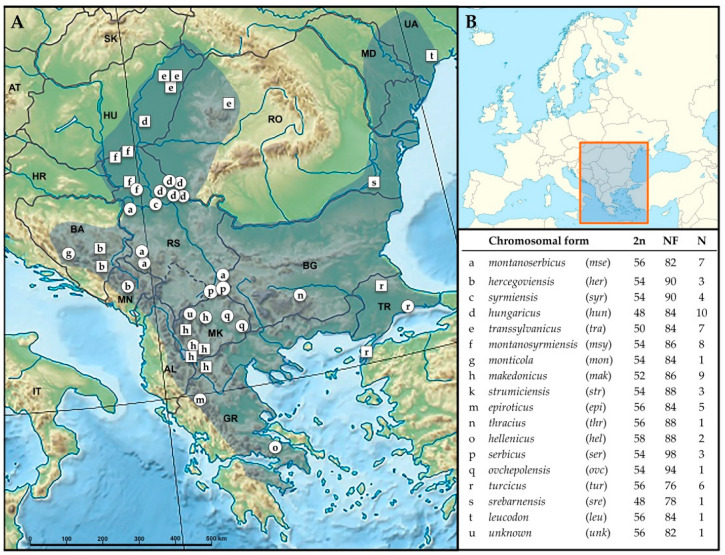
Geographic map with the distributional area of *N. leucodon* (blue shade): Circles—sampling localities of 40 accomplished sequences (IDs in Table 1); squares—sampling localities of all other imported sequences (IDs in Tables S1 and S2). Letters on the map correspond to chromosomal forms (CFs) listed herein and in Table 1, Table S1, and Table S2. N—total sample size of CFs; 2n—diploid chromosomal numbers; NF—fundamental number of chromosomal arms. (**A**)—Relief location map of Europe (source: https://commons.wikimedia.org/wiki/File:Europe_relief_laea_location_map.jpg accessed on 29 March 2022). (**B**)—Position of the study area in the European scale (source: https://d-maps.com/ accessed on 30 March 2022).

In total, 40 individuals were collected, i.e., 18 fresh tissue samples of newly captured animals from the period 2016–2021, and 22 archived teeth samples of animals caught between 1962 and 1986 (Collection Ivo Savić; IBISS) (Table 1 and Figure 1). Live individuals were treated according to Directive 2010/63/EU of the European Parliament and the Council of 22 September 2010 on the protection of animals used for scientific purposes. Following the recommendations, they were treated with anaesthesia (ketamine 27 mg/kg and xylazine 0.6 mg/kg). Because important morphometric analyses planned for future studies require skulls, the animals were euthanised. Chromosomal preparations obtained from bone marrow, using standard techniques, were performed for all specimens caught between 1962 and 1986 [16,34]. The chromosomal forms of newly captured animals were initially designated according to their distributional areas/previously described localities, as applied in [37,38,43]. Care was taken to analyse animal specimens from typical biotopes. Their CFs were additionally confirmed by phylogenetic tree clustering inferred by both DNA markers.

### 2.2. DNA Extraction and Sequencing

Liver and muscle tissues were stored in absolute ethanol or frozen at −20 °C. We extracted total DNA from this material using a DNA extraction kit (DNeasy Blood and Tissue Kit, Qiagen). DNA extraction from teeth samples was performed in a medicolegal laboratory, in which no non-human material was handled previously. The detailed procedure is described in [35], and is available from the authors upon request. Standard measures for the prevention of contamination (with human DNA) or cross-contamination (between animal samples) included bleaching, UV radiation sterilisation, and dedicated disposable tools. A highly sensitive TaqMan assay, which targets the human telomerase reverse transcriptase (hTERT) gene (Thermo Fisher Scientific), was applied to identify and remove all isolates contaminated with human DNA. The final volume of DNA extracts was in 50 µL of elution buffer. 

For amplification of ~600 bp long *16S rRNA* gene fragments, universal primers were used (Table S3), as described in [46]. The targeted regions included Domains III, IV, and partly II (position 837—1416 at *16S rRNA* gene), located between positions 1913 and 2507 of the murine mtDNA [47]. For archived samples, we applied additional semi-nested PCR, which produced 480 bp long fragments. For amplification of *MT-CYTB* gene parts, several primers were used (newly designed and modified from [48,49], Table S3) to accomplish longer sequences from DNA extracted from teeth. The reactions were set in a GeneAmp PCR System 2700 (Applied Biosystems, Waltham, MA, USA). PCR for both genes was performed with 5X Colorless GoTaq^®^ Reaction Buffer (Promega Corporation, Madison, WI, USA), 2.5 mM MgCl2, 0.4 mM of each of the dNTPs, 0.5 µM of each amplimer, 1 U of GoTaq^®^ DNA Polymerase (Promega), and 100 ng of genomic DNA, in a final volume of 50 µL. The temperature profiles were as follows: for *16S rRNA* initial denaturation (at 94 °C for 4 min), 40 cycles (94 °C for 30 s; 60 °C for 30 s; and 72 °C for 1.5 min), and a final extension at 72 °C for 7 min; for *MT-CYTB* gene initial denaturation (at 95 °C for 3 min), 35 cycles (95 °C for 30 s; 56 °C for 30 s; and 72 °C for 1 min), and a final extension at 72 °C for 3 min. Sequences were provided in both directions by a third party (Eurofins Genomics, Macrogen Europe). 

### 2.3. Data Collection and Sequence Analysis

We visually examined all sequences using the FinchTV 1.4.0 chromatogram viewer (Geospiza Inc.), and analysed them with BioEdit Ver. 7.2.5 [50], checked for the presence of stop codons and chimeric sequences, and compared with those currently available in GenBank using Basic Local Alignment Search Tool (BLAST) analysis. They were subsequently aligned using ClustalW implemented in MEGA Ver. X software [51]. 

For further *16S rRNA* analysis, 22 individual sample sequences were selected (Table 1), plus 14 reported in [35,45], all belonging to 15 *N. leucodon* CFs. An additional 33 *16S rRNA* sequences imported from GenBank (Table S1) included 7 *N. leucodon*, 9 *N. xanthodon*, 12 *N. ehrenbergi*, and 3 *Spalax* sp. sequences, as well as two of the Oriental bamboo rat *Rhizomys sinensis* subfamily Rhizomyinae (Winge, 1887) from the same family Spalacidae [52], used as an outgroup. Therefore, the final *16S rRNA* dataset had 69 individual sequences. For *MT-CYTB* phylogenetic analysis, we acquired 38 individual *N. leucodon* sample sequences (Table 1), and added 55 from GenBank (Table S2): 26 *N. leucodon*, 11 *N. xanthodon*, 8 *N. ehrenbergi*, 8 *Spalax* sp., and 2 *R. sinensis* as an outgroup. The final *MT-CYTB* dataset covered 93 individual sequences. Thus, overall, 17 *N. leucodon* CFs were analysed (*hungaricus*, *syrmiensis*, *montanosyrmiensis*, *montanoserbicus*, *serbicus*, *makedonicus*, *monticola*, *hercegovinensis*, *epiroticus*, *strumiciensis*, *hellenicus*, *ovchepolensis*, *thracius*, *turcicus*, *srebarnensis*, *transsylvanicus*, and *leucodon*; Figure 1). The geographic distribution of sampling localities of all specimens (sequences) analysed herein (Table 1, Tables S1 and S2) is given in Figure S1. 

### 2.4. Genetic Diversity and Phylogenetic Analyses

Estimation of genetic diversity parameters (*h*- number of haplotypes, *Hd*- haplotype (gene) diversity, *Pi*- nucleotide diversity, the number of fixed differences and shared mutations, a total number of polymorphic/monomorphic sites, the total number of mutations, and parsimony-informative sites) was performed in DnaSP Ver. 6 [53]. Evolutionary analyses were carried out in MEGA X using the maximum composite likelihood model for the estimation of evolutionary divergence over sequence pairs between groups with 10,000 bootstraps. They were set at two levels: between different chromosomal forms of the *N. leucodon* species complex, and between superspecies and outgroups (*Spalax* species and *R. sinensis*). Evolutionary divergences were determined using the Kimura 2-parameter model [54]. The rate of variation between sites was modelled with a gamma distribution (shape parameter = 1).

Phylogenetic analysis was performed with two different approaches to check the strength of the tree topology: the Bayesian analysis in MrBayes [55], and the maximum likelihood (ML) in PhyML [56]. The trees were created using FigTree Ver. 1.3.1 (http://tree.bio.ed.ac.uk/software/figtree/) (accessed on 29 March 2022) and MEGA X. Before phylogenetic analysis, a best-fit substitution model in aligned sequences with the Bayesian information criterion (BIC) was tested using jModelTest v.2.1.4. [57,58]. The BI analyses originated with random starting trees, and were run for 1 × 10^6^ generations, sampling every 100th generation, with the burn-in value set to 500. Combined trees of the various runs produced a 50% majority rule consensus tree with the Bayesian posterior probability values of the relevant branches. A timetree was inferred by applying the RelTime method [59,60] to the user-supplied phylogenetic tree, whose branch lengths were calculated using the maximum likelihood (ML) method and the GTR substitution model [61]. The timetree was computed using 3 calibration constraints: (1) the separation of two monophyletic genera *Spalax*/*Nannospalax* during the dry Late Miocene (6.5–8.7 mya); (2) the separation of *N. ehrenbergi* from *N. xanthodon* and *N. leucodon* (3.5–5.9 mya); and (3) the divergence from the common ancestor of *N. xanthodon* and *N. leucodon* that occurred 1.9–3.4 mya [38]. The method of Tao et al. [62] was used to set minimum and maximum time boundaries on nodes for which calibration densities were provided. All positions with less than 95% site coverage were eliminated, i.e., fewer than 5% alignment gaps, missing data, and ambiguous bases were allowed at any position (partial deletion option). Timetree analysis was conducted in MEGA X.

## 3. Results

### 3.1. 16S rRNA Gene Nucleotide Sequence Comparison

Sequences from fresh tissues and archived samples were submitted to the GenBank database under accession numbers OM691460–OM691479 and OL348372–OL348376 (Table 1). The total *16S rRNA* dataset analysed in this work contains 69 sequences, free of stop codons, insertions, or deletions. There are 112 polymorphic and 288 invariable sites. Sequence conservation C = 0.701 with three conserved regions (positions 44–124; 66–245 and 398–584). The total number of mutations was 146, out of which 96 were found to be parsimony-informative. The overall number of haplotypes h = 44; haplotype diversity Hd = 0.9795 ± 0.007; nucleotide diversity (per site) Pi = 0.0607 ± 0.0047. Evolutionary divergence analysis involved 43 *N. leucodon* sequences in a dataset with 583 positions. The overall range of estimated evolutionary divergences among 17 CFs of *N. leucodon* was between 0.003 and 0.065 (Table 2). 

The chromosomal forms *turcicus* (2n/NF = 56/76) and *thracius* (2n/NF = 56/88) stand out from other CFs, with divergence values in the highest ranges of 0.031–0.065 and 0.035–0.051, respectively. The seven CFs with demonstrated reproductive isolation ranged from 0.005 to 0.046. The evolutionary divergences between *Nannospalax* superspecies and the outgroups (*Spalax* species and *R. sinensis*) are shown in Table 3. Divergence among three species of the genus *Spalax* is between 0.041 and 0.045; between three *Nannospalax* superspecies it is 0.066–0.087 (with the lowest divergence being N. leucodon – *N. xanthodon*); and between the different genera *Spalax* and *Nannospalax* it is 0.087–0.115.

**Figure 2:**
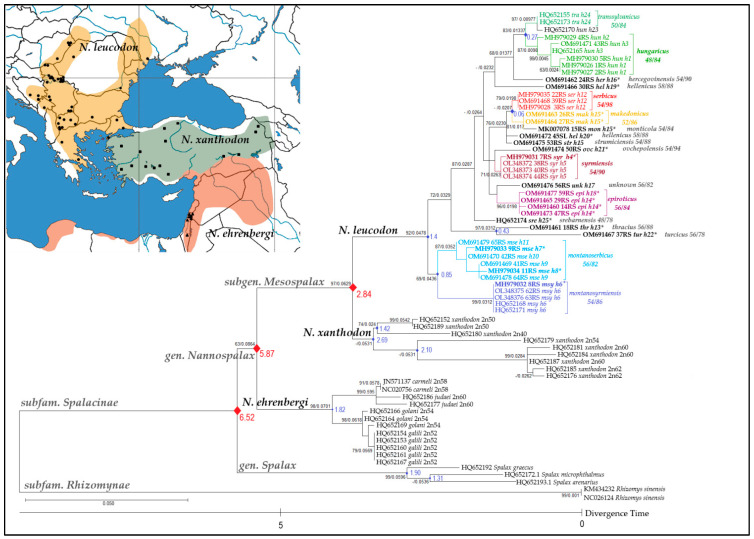
ML phylogenetic tree inferred from *16S rRNA* nucleotide comparison. Since both methods (ML and BI) produced highly similar tree topologies, support values are placed at the nodes in that order. The dash indicates branch support < 60. Red dots—calibration constraints used in the timetree analysis; blue dots—estimated divergence time. Sequences in bold with asterisks—karyotyped samples. Geographic map shows the distributional areas of three *Nannospalax* superspecies with sampling localities of all studied sequences (listed in Table 1, Tables S1 and S2, and presented in Figure S1). Source of the map: https://d-maps.com/(accessed on 29 March 2022).

The best-fit substitution model in aligned sequences, tested with jModelTest v.2.1.4., is TIM2 + I + G, and the next one listed, applied in MrBayes software, is the general time-reversible evolutionary model with a proportion of invariable sites and a gamma-shaped distribution of rates across sites (GTR + I + G). For inferring the evolutionary history via the ML method, we used the same model. The tree (Figure 2) with the highest log-likelihood (−2832.47) is shown. The percentage of trees in which the associated taxa clustered together is shown next to the branches. A discrete gamma distribution was used to model evolutionary rate differences between sites (five categories (+G, parameter = 0.3050)). The rate variation model allowed for some sites to be evolutionarily invariable ([+I], 34.99% of sites). The tree was drawn to scale, with branch lengths measured by the number of substitutions per site. This analysis involved 69 nucleotide sequences. There were a total of 583 positions in the final dataset. 

The estimated log-likelihood value of the timetree analysis was −2469.05. A discrete gamma distribution was used to model evolutionary rate differences between sites (five categories (+G, parameter = 0.3097)). The rate variation model allowed for some sites to be evolutionarily invariable ([+I], 35.07% of sites). This analysis involved 69 nucleotide sequences. All positions with less than 95% site coverage were eliminated, i.e., fewer than 5% alignment gaps, missing data, and ambiguous bases were allowed at any position (partial deletion option). There were a total of 499 positions in the final dataset. The estimated divergence times are presented in Figure 2. According to analysis of the *16S rRNA* dataset, *serbicus* and *makedonicus* CFs diverged 0.06 mya, *hungaricus* and *transsylvanicus* 0.27 mya, *thracius* and *turcicus* CFs 0.43 mya, and the whole branch of the evolutionarily oldest *N. leucodon* CFs *montanoserbicus* and *montanosyrmiensis* appeared 1.4 mya, and differentiated from one another 0.85 mya. 

The two phylogenetic methods—ML and Bayesian Inference (BI)—produced highly similar tree topologies, and the ML tree is presented in Figure 2, with the significance of both methods shown above the nodes. The phylogenetic tree depicts sister positions of *N. xanthodon* and *N. leucodon*. *N. ehrenbergi* is divided into two clades: the first clade with higher 2n, containing *N. carmeli* (2n = 58) and *N. judaei* (2n = 60); and the second, with lower 2n, containing *N. golani* (2n = 54) and *N. galili* (2n = 52). Further clustering of each of these four CFs/species is highly supported. The division of *N. xanthodon* CFs demonstrated a similar pattern, i.e., CFs with the highest 2n (60 and 62) were grouped in the basis, while CFs with the lowest 2n (40 and 50) were separated, with lower support. 

The *N. leucodon* superspecies subtree splits into two main clusters: the *montanosyrmiensis* (2n/NF = 54/86)/*montanoserbicus* (2n/NF = 56/80) clade holds a basal position. All five samples of *montanosyrmiensis* CF belong to the same haplotype (h6), although they originated from different populations (Subotička peščara and Fruška Gora Mt.) and different sampling periods. The *montanoserbicus* CF comprises six samples from a broad distributional area (Figure 1). They were collected at high altitudes of > 1000 m above sea level (a.s.l.)—Vlasina Lake (h7, h8), Zlatibor Mt. (h7), and Jadovnik Mt. (h10)—but also at lower altitudes at 63 m a.s.l., i.e., Mačvanski Pričinović (h11).

The second *N. leucodon* cluster contains *turcicus* (2n/NF = 56/88) (h13) and *thracius* (2n/NF = 56/78) (h22) in the basal position. These two CFs display the greatest genetic distances from all other CFs of the European BMR. Geographically, the most distant CF, from Bulgaria—*srebarnensis* (2n/NF = 48/78) (h25)—is also separated from the rest of the lineages, which are divided into two groups: the first is composed of CFs from the southern part of the *N. leucodon* range (except *syrmiensis*)—*epiroticus*, *ovchepolensis*, *strumiciensis*, *hellenicus*, *makedonicus*, and *serbicus*. All four *epiroticus* (2n/NF = 56/84) 16S rRNA sequences from Northern Greece clearly cluster together, with two haplotypes (h14 and h18). A sample of the unknown CF (2n/NF = 56/82) from Popova Šapka (1700 m a.s.l.) at the Šar-Mountain in North Macedonia is adjacent to *epiroticus*, but does not belong to the *montanoserbicus* CF as formerly designated in [16]. The next cluster is composed of *syrmiensis* CF (2n/NF = 54/90). A sample from Banovo Brdo (Belgrade) shows a different haplotype (h4) compared to the other three *syrmiensis* samples (h5), because of a minor chromosomal change. *Ovchepolensis* (h21) from the central part of North Macedonia is represented by only one 16S rRNA sequence, and is the most closely related to *syrmiensis*, probably because of the karyotypic similarity (2n/NF = 54/94), despite a great geographic distance. The next clade is composed of three geographically distant CFs—*makedonicus* (2n/NF = 52/86), *strumiciensis* (2n/NF = 54/88), and *monticola* (2n/NF = 54/84), which share the same haplotype (h15). All three samples of *serbicus* CF (2n/NF = 54/98) are grouped, and belong to the same haplotype (h12). 

The *hellenicus* CF (2n/NF = 58/88) from southern Greece is represented by two individual sequences, which do not cluster together. One (h20) is grouped with the heterogeneous cluster described above, and the other (h19) is placed (although questionably, with very weak support) in the basal position of the last group—*hercegovinensis*, *hungaricus*, and *transsylvanicus*. There are five haplotypes among *hungaricus* (2n/NF = 48/84) samples, divided according to populations/localities. Haplotypes h1 and h3 belong to several locations in close proximity (Deliblato, Šušara, Kajtasovo); h3 is from Vršački Breg, while h2 is from Pančevo near Belgrade. Finally, haplotype h23 from Hungary clusters together (support > 97%) with another Hungarian CF—*transsylvanicus* (2n/NF = 50/84).

### 3.2. MT-CYTB Gene Nucleotide Sequence Comparison 

This set of sequences was submitted to the GenBank database under accession numbers OM714859–OM714896 (Table 1). The total *MT-CYTB* gene dataset contains 93 sequences, also free of stop codons, insertions, and deletions. In the 802 bp sequence, 351 sites are variable (polymorphic), and 183 are invariable (monomorphic). Sequence conservation C = 0.562, with only one conserved region (positions 486–571). The total number of mutations is 188, out of which 120 were found to be parsimony-informative. The overall number of haplotypes h = 53; haplotype (gene) diversity Hd = 0.982 ± 0.005; nucleotide diversity (per site) Pi = 0.0922 ± 0.0073.

The *MT-CYTB* dataset involved 63 *N. leucodon* sequences and a total of 802 positions. Evolutionary divergence analysis revealed generally higher values compared to *16S rRNA*. The overall range among 17 CFs of *N. leucodon* was between 0.008 and 0.111 (Table 4). *Montanoserbicus* and *montanosyrmiensis* have divergence values against all other CFs in the highest ranges, at 0.071–0.108 and 0.071–0.111, respectively. The seven CFs with demonstrated reproductive isolation ranged between 0.017 and 0.104. Divergences between all three *Nannospalax* superspecies and the outgroups are presented in Table 5. Five species of the genus *Spalax* diverged between 0.055 and 0.131, and the three *Nannospalax* superspecies diverged between 0.100 and 0.129 (also with the lowest divergence between *N. leucodon* and *N. xanthodon*), while between the different genera *Spalax* and *Nannospalax* the divergence was 0.185–0.218. 

**Figure 3:**
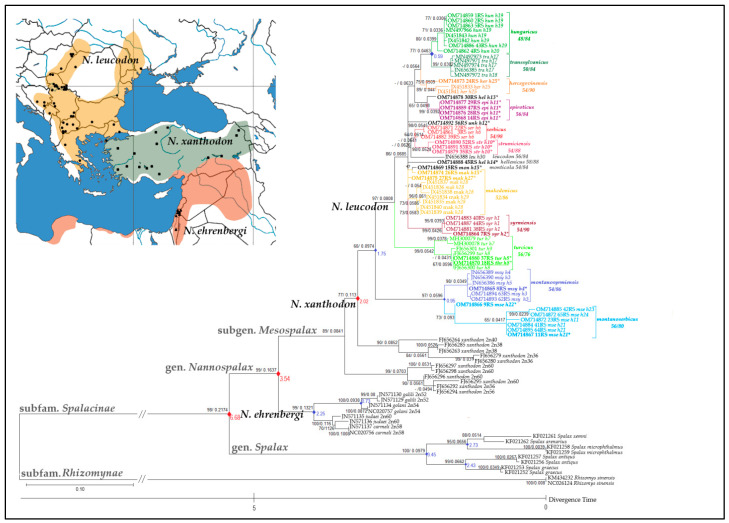
ML phylogenetic tree inferred from *MT-CYTB* nucleotide comparison. Since both methods (ML and BI) produced highly similar tree topologies, support values are placed at the nodes in that order. The dash indicates branch support < 60. Red dots—calibration constraints used in the timetree analysis; blue dots—estimated divergence time. Sequences in bold with asterisks—karyotyped samples. Geographic map shows distributional areas of the three *Nannospalax* superspecies, with specified sampling localities of all studied sequences (listed in Table 1, Tables S1 and S2, and presented in Figure S1). Source of the map: https://d-maps.com/ (accessed on 29 March 2022).

According to jModelTest v.2.1.4., the best-fit substitution model in the aligned sequences tested was TVM + I + G. The ML tree is presented in Figure 3, with the significance of both methods shown above the nodes. The tree with the highest log-likelihood (−5868.10) is shown. The percentage of trees in which the associated taxa were clustered together is shown next to the branches. A discrete gamma distribution was used to model evolutionary rate differences between sites (five categories (+G, parameter = 0.4196)). The rate variation model allowed for some sites to be evolutionarily invariable ([+I], 28.24% of sites). The final dataset involved 93 nucleotide sequences and a total of 802 positions. 

The estimated log-likelihood value of the tree was −3110.51. A discrete gamma distribution was used to model evolutionary rate differences between sites (five categories (+G, parameter = 1.5465)). The rate variation model allowed for some sites to be evolutionarily invariable ([+I], 52.47% of sites). This analysis involved 93 nucleotide sequences. The codon positions included were the 1st + 2nd + 3rd + noncoding. All positions with less than 95% site coverage were eliminated, i.e., fewer than 5% alignment gaps, missing data, and ambiguous bases were allowed at any position (partial deletion option). There were a total of 415 positions in the final dataset. According to analysis of the *MT-CYTB* dataset, the evolutionarily oldest *montanoserbicus* and *montanosyrmiensis* CF branches of *N. leucodon* appeared 1.75 mya, and differentiated from one another 0.95 mya, while the *hungaricus* and *transsylvanicus* CFs diverged mutually 0.59 mya (Figure 3).

*N. ehrenbergi* and *N. xanthodon* followed the same clustering pattern into two groups—with lower and higher 2n. As demonstrated from the *16S rRNA* dataset, *N. ehrenbergi* is separated into clades of *N. carmeli/N. judaei* with higher 2n (58/60) and *N. galili/N. golani* with lower 2n (54/52). *N. xanthodon* CFs were distributed as follows: CFs with higher 2n (56/60) were grouped in the basis, and CFs with the lowest 2n (36, 38, and 40) were closer to the *N. leucodon* cluster. The *MT-CYTB* phylogenetic tree topology (Figure 3) follows the same pattern as that of *16S rRNA*, i.e., the *N. leucodon* subtree splits into two main clusters. The *montanosyrmiensis*/*montanoserbicus* clade holds a basal position, and gathers together with the highest support. Contrary to only one haplotype in *16S rRNA*, the six samples of *montanosyrmiensis* in the *MT-CYTB* analysis exhibit three haplotypes distributed regardless of population affiliation (e.g., h3 is present in both populations: Kelebia and Sremski Karlovci) and different sampling periods (e.g., h4 was sampled in 1965 and 2013). Another cluster composed of seven *montanoserbicus* samples holds four haplotypes. Haplotype h21 is located exclusively in Vlasina (four samples collected in 1965 and 2019–2021). Two samples from lower altitudes are grouped (h23 and h24). One animal from Zlatibor Mt. (h22) is separated from the whole group, but still in the cluster of the same CF.

The second *N. leucodon* clade is heterogeneous, and again contains *turcicus* in the basal position. Six samples from three localities revealed three haplotypes: h7 (two samples from Eceabat), h8, h9 (Kırklareli), h8 (Çorlu) and, finally, the same haplotype h8 described as another *thracius* CF. The evolutionary distance between these CFs is also extremely low (0.008). The next cluster is *syrmiensis* CF, which holds two haplotypes: again h2 from Banovo Brdo, separated from three other samples (h1). Seven imported *makedonicus* sequences from Bistra (h29, h28), Pelister (h28), and Galičica (h28) all group together, while samples from Ohrid (h27) and Jakupica (h15) are slightly separated. *Monticola* CF from Bosnia and Herzegovina is gathered with the last one—the same haplotype h15. Three samples of each CF—*strumiciensis* (h10) and *serbicus* (h6)—segregate separately. An animal from Popova Šapka of unknown *N. leucodon* CF is placed between *serbicus* and *epiroticus*. All four *epiroticus* samples represent the same h11 haplotype. As in the case of *16S rRNA* data, one sample from Southern Greece (*hellenicus* CF, h13) is grouped closer to *epiroticus* from Northern Greece, and another *hellenicus* (h14) is closer to the *strumiciensis*/*serbicus* clade. The last subtree contains three clades: first, *hercegovinensis* CF, with two haplotypes (h25 and h26); second, *hungaricus* CF (one sample h19, and seven samples h20); and third, *transsylvanicus* CF, comprising haplotypes h17, h18, and h19. 

## 4. Discussion

Here, we analyse the nucleotide polymorphism of two functionally different mitochondrial genes with distinct mutational rates, to infer the extent of molecular diversification that follows intense chromosomal rearrangements and resolve taxonomical difficulties within the European BMR *N. leucodon* species complex. Our results show that both genes are suitable molecular markers for the delineation of the majority of *N. leucodon* CFs/cryptic species. However, their resolving power varies and, as expected, *MT-CYTB* analysis commonly detects more haplotypes and produces higher evolutionary divergences. In only two CFs—*epiroticus* and *hungaricus*—was the *16S rRNA* marker more resolute than the *MT-CYTB* marker. Out of 17 examined CFs, 11 (with *MT-CYTB*) and 9 (with *16S rRNA*) clearly cluster into discrete groups with high support, in a similar fashion in all phylogenetic trees. The segregation into separate clusters could be expected based on previous findings [35,36]. However, two earlier studies reported lower genetic differentiation among *N. leucodon* CFs compared to the other two superspecies [37,38]. This can be explained by inadequate sample sizes—i.e., the number of analysed individuals and CFs—and/or by incorrect labelling of CFs. In our dataset, six CFs (*srebarnensis*, *hellenicus*, *monticola*, *ovchepolensis*, *leucodon*, and *thracius*) were represented with only one or two samples. For that reason, their phylogenetic status remains unresolved with both markers. Another reason may be recent divergence times, i.e., some of these CFs diverged recently, presenting lower genetic differentiation and an imprecise phylogenetic state; however, these were below the detection level in our timetree analysis. 

It was hypothesised that *N. leucodon* CFs possibly emerged from a common initial ancestor through a rapid chromosomal evolution within several parallel courses [17,63]. The whole speciation of the recent Balkan BMRs took place during the Pleistocene, when a variety of karyotypes developed as a result of their adaptation to diverse environments during the process of allopatric speciation. Throughout the dry Late Miocene (~7.6 Ma), a marine barrier between Anatolia and the Balkans emerged, and the two groups *Spalax* and *Nannospalax* differentiated [38,64]. The topologies of our phylogenetic trees inferred from both datasets are consistent with the traditional taxonomy of recent BMRs, i.e., monophyletic origin of two genera: the greater BMR *Spalax*, and the lesser BMR *Nannospalax* [22,37,38,44,52,65,66]. It is interesting that, unlike *Nannospalax*, species of the genus *Spalax* are morphologically/ecologically distinguished, and have conserved karyotypes with high 2n (60–62) [44]. Since BMRs from both genera share the same fossorial lifestyle, extreme environmental conditions may have an impact on stabilising the selection of morphology [1], but are probably not a cause of intensive chromosomal speciation in the genus *Nannospalax*. Environmental influence, overall mutation rates, population sizes, and the activation of mobile elements and retroviruses were described as possible factors that contribute to the rate of karyotypic evolution in mammals [67]. Furthermore, our results prove that the lesser BMR is divided into two subgenera, as proposed by [22,65]: *Nannospalax* s. str., comprising only one superspecies (*N. ehrenbergi*), and *Mesospalax*, with *N. xanthodon* and *N. leucodon* superspecies. This was also confirmed by evolutionary distances calculated from both datasets, with the lowest values among the latter two superspecies.

### 4.1. Delineation of 11 N. leucodon CFs

Phylogenetic analysis of both genes enabled clear outlining of a total of 11 CFs into separate clusters. Tree topology was consistent with our previous findings obtained from *16S rRNA* data [35], and was additionally confirmed here with a considerably larger number of CFs with the *MT-CYTB* dataset. Moreover, the topology was in accordance with the phylogeny of several *N. leucodon* CFs reported in [36,38,68]. The results from both datasets, in the basal position of *N. leucodon* phylogenetic subtrees, placed two completely allopatric CFs (2n = 54 and 2n = 56) each in a separate cluster: *montanosyrmiensis* and *montanoserbicus*. Although their 2n differs in two chromosome pairs, the karyotype morphology is significantly different (for details see [35]), and they were proven to be reproductively isolated [16,34,35]. Their mutual divergence time is estimated at 0.85/0.95 mya by *16S rRNA* and *MT-CYTB* data, respectively. Their basal position in phylogenetic trees is also substantiated by high evolutionary divergence values that overlap with those recorded among higher taxonomic categories, pointing to an earlier divergence (1.4/1.75 mya, as proposed by *16S rRNA* and *MT-CYTB*, respectively) compared to other CFs. The *montanosyrmiensis* CF was initially described as the resident of the steppe habitats of the foothills of Mt. Fruška Gora, in the southern edge of the Pannonian Plain, Serbia [16]. Nevertheless, recent publications reported fragmented distributional area for four additional populations: one in Serbia (Kelebia), and three isolated populations in Hungary, all estimated as endangered or critically endangered [36,43]. The *montanoserbicus* CF was described as the resident of a wide, discontinuous area of island-type isolated habitats of xeromontano steppe in the preglacial Mid Balkan Mountains (North Dinarides, Rila–Rhodope, and the Balkan system) above 700 m a.s.l. [16]. However, present data from Mačvanski Pričinović, near Bogatić, report this CF at only 63 m a.s.l.—the locality formerly assigned as the western border of *syrmiensis*’ area [16]. Considering a wide distribution, *montanoserbicus* is not labelled as threatened, but the populations should be carefully monitored. 

According to both datasets, the *turcicus* CF (Makri-Koi Istanbul, Turkey) clusters together with only one sample of *thracius* (Novo Selo, the Thracian Plain, Bulgaria). Despite the fact that more samples were available for *MT-CYTB* analysis, the *16S rRNA* dataset reveals different haplotypes, and estimates the divergence time between these two CFs at 0.43 mya. They both inhabit arable steppe with a deep layer of soil on the Thracian Plain, and may even spread towards one another. While they share the same 2n = 56, their chromosomal morphology differs, especially in acrocentric and subacrocentric groups. However, both CFs produced high molecular discrepancies towards other *N. leucodon* CFs. More data from the field on their recent distribution and potential hybrid zones are necessary. 

*Syrmiensis* CF inhabits a small distributional area: Belgrade region, Avala Mt., Srem, and Mačva (the steppe habitats of the Pannonian Plain and the ancient Pannonian shoreline—the lower belt) [16]. Crossbreeding experiments with six other CFs demonstrated reproductive isolation. Recently, this CF was declared extinct, because there were no data on it since 1983 [36,68,69]. Even if it is still present in some localities, its distributional area is highly reduced. Therefore, it is not extinct, but critically threatened [45]. 

Both datasets revealed close relationships of three CFs that occupy the southeastern and far southern parts of the Balkan Peninsula [5,16,70]: *makedonicus* and *strumiciensis* from North Macedonia, and *serbicus* from Serbia. They have a parapatric distribution and a similar 2n (52, 54). The *strumiciensis* CF is described as a resident of steppe habitats in the border regions of the territories of the Macedonian hilly and piedmont area, as well as the Rila–Rhodope mountain system. It was proposed that the area possibly spreads to the southern and eastern parts of North Macedonia, along the Strumica River, towards the territories of Greece and Bulgaria, but more new data from the field are required. The *serbicus* CF resides in the broadest area from the Danube near Ram and Djerdap Gorge, southwards down the Great Morava Valley, the Timok and South Morava Rivers, and the Vardar River to the town of Veles in North Macedonia, Kosovo in the west, and up to the town of Pirot to the east. The *makedonicus* CF inhabits a wide range of altitudes from above 2200 m a.s.l. at Mt. Jakupica to lower elevations in Pelagonia (between the towns of Prilep and Bitola) and in the Lake Ohrid Basin, as well as in the valleys of Northern Greece. Both markers in this study succeeded in delineation of these CFs into three separate groups. The *makedonicus* and *serbicus* CFs are reproductively completely isolated from one another [16,34,35], and their estimated divergence time by *16S rRNA* is 0.06 mya, which suggests their recent diversification. The *epiroticus* CF inhabits the rocky semi-desert of Epirus, high altitudes in Makedonia, and lower altitudes in Pelagonia, the Lake Ohrid Basin, and the valleys of Northern Greece. All four *epiroticus* specimens cluster together, with the highest support. 

The last cluster includes three evolutionarily younger CFs with similar karyotypes [17,63]: *hungaricus*, from a large area of Pannonian lowland in Bačka, Banat, and the narrow belt in the north of Serbia; *transsylvanicus*, distributed in northwestern Romania [71] and eastern Hungary [69], which diverged from the former 0.27/0.59 mya, as proposed by *16S rRNA* and *MT-CYTB*, respectively; and the most closely related to them, the *hercegovinensis* CF—a resident of the xeromontano steppe habitats of the Montenegro/East Bosnian Mountains of the Dinaric system (Mt. Čemerno and the Neretva River, Mt. Gvozd). All three CFs are clearly delineated by both markers into distinct clades, and express low values of evolutionary divergence, confirming their recent diversification [17,63]. 

### 4.2. Chromosomal Speciation, Reproductive Isolation, and the Biological Species Concept

Chromosomal rearrangements as a speciation mechanism have been carefully studied [32,72,73,74], although there are not many described cases [33,75,76,77,78]. Even though chromosomal modifications may consequently alter organisms’ morphology and/or physiology, this is not always necessary. One explanation would be that Robertsonian fusions and fissions, as the most frequent chromosomal changes, do not modify the genome size or qualitatively affect its genetic significance [79]. As chromosomal differences accumulate, the fertility and viability of hybrids gradually decrease due to meiotic difficulties, thereby reducing the gene flow [32,33,74,80]. After complete reproductive isolation, nucleotide mutations may accumulate (detected as higher genetic divergence values). after which the genomes evolve independently. 

Among the 11 distinguished CFs discussed above, 7 (with sympatric and parapatric distribution) are already documented as being completely reproductively isolated: *montanosyrmiensis*, *syrmiensis*, *montanoserbicus*, *serbicus*, *makedonicus*, *hungaricus*, and *monticola*. The degree of karyotypic divergences (in 2n and/or NF) between CFs that were not involved in crossbreeding experiments—i.e., *epiroticus*, *strumiciensis*, *hercegovinensis*, and *transsylvanicus*—corresponds to those reproductively isolated [16,34,35]. Specifically, it was shown that CFs with identical 2n but different NF (e.g., 2n = 54 in *syrmiensis, serbicus, monticola,* and *montanosyrmiensis*) failed to produce embryos. Therefore, we can presume reproductive isolation for these four CFs as well. To verify this, more fieldwork for detecting eventual natural hybrids and/or crossbreeding experiments must be accomplished. Until now, natural hybrids had not been found among *N. leucodon* CFs [16]; they are seemingly absent or very infrequent in Anatolia [34,81,82,83,84], and are commonly described only in Israel [24]. In addition to reproductive isolation and specific ecological characteristics [11,16], additional criteria for cryptic species delineation include the evolutionary divergence values observed herein and in previous studies [35,36]. It is noticeable that divergences between *N. leucodon* CFs inferred with both markers overlap with acknowledged higher taxonomic categories (*Spalax* species), or sometimes exceed them—especially *montanosyrmiensis*, *montanoserbicus*, *turcicus*, and *thracius* CFs. These forms are characterised by a higher 2n (54 and 56) compared to other examined *N. leucodon* CFs. The fact that CFs with higher 2n are evolutionarily older corresponds to the fusion hypothesis of *N. leucodon* karyotype evolution, i.e., Robertsonian rearrangements most likely acted in the direction of a decrease in the number of acrocentric autosomes and 2n [16,17]. The monophyletic origin of the two genera *Spalax* (2n = 60–62) and *Nannospalax* further supports this presumption.

The divergences between some *N. leucodon* CFs determined using *MT-CYTB* markers even correspond to those between the three *Nannospalax* superspecies, pointing to their probable cryptic species status. When the problem of conservation of cryptic species appears, it is important to apply suitable species definition for their classification [4]. The application of the biological species concept (BSC) is hardly ever appropriate, and can illuminate only a small number of species [85], but is highly suitable for conservation concerns [4]. Here, we have a rare opportunity to apply the BSC, acknowledging seven reproductively isolated *N. leucodon* CFs as cryptic species in order to implement conservation measures and preserve biological diversity.

### 4.3. Major Factors of Threat and Implications for Conservation

Along with changes in habitat—especially in the last 100 years, due to anthropogenic effects—the distributional area of the European BMR species complex *N. leucodon* has been gradually reduced, so that whole populations have disappeared [43,68]. According to paleontological data, they once occupied areas of present-day Germany, Austria, Poland, Western and Central Hungary, Southern Greece, and the Aegean islands [22,86]. BMRs are long-lived rodents that live in dry grassland habitats, spending their whole lives in underground tunnel systems, and feeding mostly on roots, tubers, rhizomes, grass, etc. Despite the official strictly protected status of the whole *N. leucodon* superspecies in the Republic of Serbia, as proposed by [87], the natural habitats of Serbian *N. leucodon* cryptic species have been persistently reduced, largely because of invasive agriculture and massive urbanisation. They are thus forced to migrate closer to human settlements, gardens, farms, and parks, where they are treated as pests and killed because they cause material damage. By unnecessary killing, humans significantly increase the negative pressure on the number of individuals and their populations [16,43]. According to the IUCN-CMP Unified Classification of Direct Threats [88], the main risk factors that affect the decline in the number of individuals in populations, their mutual isolation, and in some cases complete extinction are as follows: urbanisation and infrastructure construction (1.1 and 1.2); formation of agro-ecosystems and transition to intensive agricultural production (2.1.1 and 2.1.3); road construction (4.1); burning of vegetation (7.1); systematic transition from small-scale extensive production to large-scale intensive production systems (7.3); and habitat pollution (9.2 and 9.3). Necessary protection measures, also according to the IUCN-CMP Unified Classification of Conservation Actions Needed [89], are as follows: assessing the population structure of cryptic species with reduced area; and preserving their gene pool to avoid genetic bottleneck through the introduction of relocation programs (3.1 and 3.3). Additionally, it is necessary to advise the local residents about the level of protection of BMRs, and ways to protect their households without killing the animals (4.3); increase the competence and efficiency of existing protection and monitoring services; update the protected species lists (5.4); form and fund scientific teams (international cooperation) to raise the level of research of insufficiently studied taxa and areas (7.3); and establish new protected areas.

## 5. Conclusions

Intense chromosomal rearrangements in the European lesser BMR have produced many lineages with karyotypes changed by sufficient amounts to drive meiotic difficulties, reduced gene flow and, eventually, reproductive isolation, as demonstrated through previous crossbreeding experiments among seven chromosomal forms (CFs). Therefore, the BSC may be applied for classification to conserve their biodiversity. Although those investigations included a limited number of *N. leucodon* CFs, according to the results presented herein, it can be expected that 11 CFs delineated in the phylogenetic trees may be reproductively isolated as well. Therefore, more fresh samples and additional nuclear gene markers are necessary to determine their cryptic species status—needed for participation in conservation strategies. Additionally, CFs with higher 2n exhibit evolutionary divergence values typical of officially accepted species, as supported by divergence time estimates. These findings substantiate the fusion hypothesis of *Nannospalax* karyotype evolution. As the distributional areas vary, the estimation of the risk levels and conservation actions should begin with those that have significantly reduced and/or fragmented distributions. Here, we draw particular attention towards two CFs/cryptic species estimated as endangered and critically endangered: *syrmiensis*, and *montanosyrmiensis*. For other possible cryptic species delineated by mtDNA genes in this study, detailed monitoring and population ecology surveys are necessary.

## Figures and Tables

**Table 1:** A list of 40 *N. leucodon* samples, with IDs (GenBank accession numbers) for mtDNA gene sequences. IDs shown in bold were published previously [35,45]. 2n—diploid chromosomal numbers; NF—fundamental number of chromosomal arms. See Figure 1 for abbreviations of chromosomal forms and their sampling localities (letters in parentheses).

16S rRNA ID	MT-CYTB ID	Sample No	Sampling Locality ^b^	Chromosomal Form	2n/NF	Year of Capture	Tissue
**MH979033** *^h^* 7	OM714866 *^h^* 22	9RS ^a^	Zlatibor Mt, RS	*mse* (a)	56/82	1965	Tooth
**MH979034** *^h^* 8	OM714867 *^h^* 21	11RS ^a^	Vlasina, RS	*mse* (a)	56/82	1965	Tooth
/	OM714872 *^h^* 11	23RS	Vlasina, RS	*mse* (a)	56/82	2018	Muscle
OM691469 *^h^* 9	OM714884 *^h^* 21	41RS	Vlasina, RS	*mse* (a)	56/82	2020	Liver
OM691470 *^h^* 10	OM714885 *^h^* 23	42RS	Jadovnik Mt, RS	*mse* (a)	56/82	2020	Liver
OM691478 *^h^* 9	OM714895 *^h^* 21	64RS	Vlasina, RS	*mse* (a)	56/82	2021	Liver
OM691479 *^h^* 11	OM714896 *^h^* 24	65RS	Mačvanski Pričinović, RS	*mse* (a)	56/82	2017	Liver
OM691462 *^h^* 16	OM714873 *^h^* 25	24RS ^a^	Gvozd Mt, MN	*her* (b)	54/90	1967	Tooth
**MH979031** *^h^* 4	OM714864 *^h^* 2	7RS ^a^	Beograd, RS	*syr* (c)	54/90	1962	Tooth
**OL348372** *^h^* 5	OM714881 *^h^* 1	38SL	Beograd, RS	*syr* (c)	54/90	2019	Liver
**OL348373** *^h^* 5	OM714883 *^h^* 1	40RS	Beograd, RS	*syr* (c)	54/90	2020	Liver
**OL348374** *^h^* 5	OM714887 *^h^* 1	44RS	Beograd, RS	*syr* (c)	54/90	2021	Liver
**MH979026** *^h^* 1	OM714859 *^h^* 19	1RS	Šumarak, RS	*hun* (d)	48/84	2016	Liver
**MH979027** *^h^* 1	OM714860 *^h^* 19	2RS	Šumarak, RS	*hun* (d)	48/84	2016	Liver
**MH979029** *^h^* 2	OM714862 *^h^* 20	4RS	Pančevo, RS	*hun* (d)	48/84	2017	Liver
**MH979030** *^h^* 1	OM714863 *^h^* 19	5RS	Kajtasovo, RS	*hun* (d)	48/84	2018	Liver
OM691471 *^h^* 3	OM714886 *^h^* 19	43RS	Vršački Breg, RS	*hun* (d)	48/84	2020	Liver
**MH979032** *^h^* 6	OM714865 *^h^* 4	8RS ^a^	Stražilovo, RS	*msy* (f)	54/86	1965	Tooth
OL348375 *^h^* 6	OM714893 *^h^* 3	62RS	Sremski Karlovci, RS	*msy* (f)	54/86	2021	Liver
OL348376 *^h^* 6	OM714894 *^h^* 3	63RS	Sremski Karlovci, RS	*msy* (f)	54/86	2021	Liver
**MK007078** *^h^* 15	OM714869 *^h^* 15	15RS ^a^	Šuica, BA	*mon* (g)	54/84	1979	Tooth
OM691463 *^h^* 15	OM714874 *^h^* 15	26RS ^a^	Jakupica, MK	*mak* (h)	52/86	1972	Tooth
OM691464 *^h^* 15	OM714875 *^h^* 27	27RS ^a^	Ohrid, MK	*mak* (h)	52/86	1975	Tooth
/	OM714879 *^h^* 10	35RS ^a^	Strumica, MK	*str* (k)	54/88	1975	Tooth
/	OM714890 *^h^* 10	52RS ^a^	Strumica, MK	*str* (k)	54/88	1975	Tooth
OM691475 *^h^* 15	OM714891 *^h^* 10	53RS ^a^	Strumica, MK	*str* (k)	54/88	1975	Tooth
OM691460 *^h^* 14	OM714868 *^h^* 11	14RS ^a^	Lefkothea, GR	*epi* (m)	56/84	1975	Tooth
/	OM714876 *^h^* 11	28RS ^a^	Lefkothea, GR	*epi* (m)	56/84	1975	Tooth
OM691465 *^h^* 14	OM714877 *^h^* 11	29RS ^a^	Lefkothea, GR	*epi* (m)	56/84	1975	Tooth
OM691473 *^h^* 14	OM714889 *^h^* 11	47RS ^a^	Lefkothea, GR	*epi* (m)	56/84	1975	Tooth
OM691477 *^h^* 18	/	59RS ^a^	Lefkothea, GR	*epi* (m)	56/84	1975	Tooth
OM691461 *^h^* 13	OM714870 *^h^* 8	18RS ^a^	Novo Selo, BG	*thr* (n)	56/76–78	1976	Tooth
OM691466 *^h^* 19	OM714878 *^h^* 13	30RS ^a^	Levadia, GR	*hel* (o)	58/88	1975	Tooth
OM691472 *^h^* 20	OM714888 *^h^* 14	45RS ^a^	Levadia, GR	*hel* (o)	58/88	1975	Tooth
**MH979028** *^h^* 12	OM714861 *^h^* 6	3RS	Ristovac, RS	*ser* (p)	54/98	2014	Muscle
**MH979035** *^h^* 12	OM714871 *^h^* 6	22RS	Klinovac, RS	*ser* (p)	54/98	2018	Muscle
OM691468 *^h^* 12	OM714882 *^h^* 6	39RS	Ristovac, RS	*ser* (p)	54/98	2019	Muscle
OM691474 *^h^* 21	/	50RS ^a^	Štip, MK	*ovc* (q)	54/94	1986	Tooth
OM691467 *^h^* 22	OM714880 *^h^* 8	37RS ^a^	Çorlu, TR	*tur* (r)	56/76–78	1976	Tooth
OM691476 *^h^* 17	OM714892 *^h^* 12	56RS ^a^	Popova Šapka, MK	*unk* (u)	56/82	1972	Tooth

^a^—Collection Ivo Savic; ^b^—ISO country code; *^h^*—haplotype number.

**Table 2:** Estimates of evolutionary divergence over sequence pairs between *N. leucodon* CFs inferred from the *16S rRNA* dataset. The number of base substitutions per site from averaging over all sequence pairs between groups is shown. Standard error estimates, obtained via a bootstrap procedure (10,000 replicates), are shown above the diagonal. See Figure 1 for abbreviations of CFs.

	*hun*	*syr*	*msy*	*mse*	*ser*	*her*	*mak*	*mon*	*unk*	*epi*	*str*	*hel*	*ovc*	*thr*	*tur*	*tra*	*sre*
*hun*		0.006	0.009	0.009	0.007	0.006	0.007	0.009	0.006	0.007	0.007	0.006	0.009	0.011	0.012	0.004	0.008
*syr*	0.027		0.009	0.008	0.005	0.006	0.005	0.006	0.005	0.005	0.004	0.004	0.002	0.009	0.010	0.006	0.005
*msy*	**0.045**	**0.040**		0.006	0.010	0.010	0.010	0.012	0.008	0.009	0.009	0.009	0.012	0.010	0.011	0.009	0.009
*mse*	**0.040**	0.033	0.023		0.009	0.009	0.009	0.011	0.008	0.009	0.009	0.008	0.010	0.009	0.010	0.009	0.008
*ser*	0.025	0.014	**0.044**	0.039		0.007	0.003	0.005	0.005	0.006	0.003	0.004	0.004	0.010	0.010	0.006	0.006
*her*	0.022	0.023	**0.044**	0.037	0.027		0.008	0.010	0.006	0.007	0.007	0.006	0.009	0.011	0.012	0.006	0.006
*mak*	0.030	0.016	**0.046**	**0.041**	0.005	0.029		0.003	0.005	0.006	0.004	0.004	0.005	0.010	0.010	0.007	0.006
*mon*	0.038	0.020	**0.059**	**0.048**	0.010	0.038	0.004		0.007	0.008	0.005	0.006	0.005	0.009	0.012	0.009	0.008
*unk*	0.025	0.014	0.036	0.028	0.014	0.021	0.016	0.023		0.005	0.005	0.005	0.005	0.010	0.011	0.006	0.005
*epi*	0.029	0.015	**0.041**	0.038	0.020	0.025	0.021	0.027	0.014		0.006	0.005	0.006	0.010	0.011	0.007	0.006
*str*	0.026	0.012	0.038	0.037	0.005	0.025	0.007	0.012	0.012	0.018		0.004	0.003	0.010	0.011	0.007	0.006
*hel*	0.029	0.018	**0.044**	0.039	0.017	0.026	0.017	0.024	0.020	0.020	0.015		0.004	0.009	0.010	0.006	0.005
*ovc*	0.032	0.003	**0.050**	0.038	0.007	0.027	0.010	0.010	0.009	0.015	0.005	0.016		0.010	0.010	0.008	0.007
*thr*	**0.051**	0.035	**0.043**	0.037	0.038	**0.046**	**0.041**	0.038	**0.043**	**0.043**	**0.041**	**0.043**	0.035		0.008	0.010	0.010
*tur*	**0.065**	**0.052**	**0.056**	**0.050**	**0.052**	**0.064**	**0.050**	**0.054**	**0.056**	**0.057**	**0.054**	**0.055**	**0.037**	**0.031**		0.011	0.010
*tra*	0.012	0.021	**0.042**	0.037	0.022	0.016	0.023	0.032	0.020	0.023	0.023	0.026	0.024	**0.043**	**0.058**		0.007
*sre*	0.032	0.016	0.036	0.029	0.020	0.020	0.021	0.029	0.016	0.021	0.018	0.021	0.017	0.039	**0.050**	0.025	

Bold—values equal to those recorded among *Spalax* species; underlined—seven reproductively isolated CFs.

**Table 3:** Evolutionary divergence over sequence pairs between superspecies and outgroups inferred from the comparison of *16S rRNA* gene sequence data. Standard error estimates are shown above the diagonal.

	*N. l*	*N. x*	*N. e*	*S. g*	*S. m*	*S. a*	*R. s*
*N. leucodon*		0.009	0.013	0.016	0.016	0.017	0.022
*N. xanthodon*	0.066		0.012	0.016	0.015	0.016	0.021
*N. ehrenbergi*	0.087	0.081		0.014	0.015	0.014	0.020
*S. graecus*	0.107	0.113	0.087		0.009	0.010	0.023
*S. microphthalmus*	0.110	0.112	0.099	**0.041**		0.010	0.022
*S. arenarius*	0.115	0.118	0.094	**0.045**	**0.045**		0.022
*R. sinensis*	0.173	0.172	0.153	0.172	0.164	0.167	

Bold—values among *Spalax* species; underlined—values among three *Nannospalax* superspecies.

**Table 4:** Estimates of evolutionary divergence over sequence pairs between *N. leucodon* CFs inferred from the *MT-CYTB* dataset. Standard error estimates are shown above the diagonal. See Figure 1 for abbreviations of CFs.

	*mse*	*mak*	*tur*	*tra*	*hel*	*syr*	*her*	*msy*	*epi*	*unk*	*ser*	*hun*	*mon*	*str*	*leu*
*mse*		0.015	0.015	0.014	0.017	0.016	0.014	0.013	0.018	0.017	0.015	0.013	0.013	0.018	0.015
*mak*	**0.099**		0.010	0.007	0.006	0.009	0.006	0.014	0.008	0.008	0.005	0.006	0.004	0.007	0.008
*tur*	**0.090**	0.050		0.011	0.011	0.013	0.011	0.016	0.011	0.013	0.010	0.011	0.010	0.013	0.012
*tra*	**0.102**	0.043	**0.063**		0.008	0.010	0.007	0.015	0.009	0.011	0.008	0.006	0.008	0.009	0.009
*hel*	**0.100**	0.027	0.052	0.035		0.011	0.007	0.018	0.007	0.008	0.006	0.009	0.007	0.009	0.010
*syr*	**0.104**	0.042	**0.072**	**0.062**	0.050		0.010	0.017	0.013	0.013	0.008	0.010	0.008	0.011	0.011
*her*	**0.088**	0.031	**0.062**	0.037	0.033	0.052		0.016	0.009	0.008	0.006	0.006	0.005	0.009	0.008
*msy*	**0.071**	**0.093**	**0.094**	**0.103**	**0.100**	**0.108**	**0.102**		0.018	0.018	0.016	0.014	0.014	0.019	0.017
*epi*	**0.102**	0.028	0.049	0.031	0.025	**0.055**	0.033	**0.092**		0.009	0.009	0.010	0.008	0.011	0.012
*unk*	**0.080**	0.020	0.051	0.035	0.030	0.048	0.027	**0.081**	0.024		0.009	0.010	0.007	0.011	0.012
*ser*	**0.097**	0.022	0.050	0.044	0.024	**0.038**	0.033	**0.099**	0.031	0.025		0.007	0.005	0.006	0.007
*hun*	**0.093**	0.033	**0.062**	0.029	0.040	**0.053**	0.032	**0.092**	0.038	0.033	0.038		0.006	0.010	0.008
*mon*	**0.091**	0.017	0.049	0.041	0.026	**0.041**	0.026	**0.097**	0.027	0.018	0.019	0.029		0.008	0.007
*str*	**0.108**	0.029	**0.063**	0.045	0.036	0.053	0.040	**0.111**	0.045	0.033	0.018	0.047	0.028		0.009
*leu*	**0.108**	0.043	**0.071**	**0.056**	0.046	**0.061**	0.044	**0.118**	0.046	0.043	0.037	0.049	0.037	0.041	

Bold—values equal to those recorded among *Spalax* species; underlined—seven reproductively isolated CFs.

**Table 5:** Estimates of evolutionary divergence over sequence pairs inferred from the *MT-CYTB* dataset between three *Nannospalax* superspecies and the outgroups.

	*N. e*	*N. l*	*S. z*	*N. x*	*S. a*	*R. s*	*S. ar*	*S. m*	*S. g*
*N. ehrenbergi*		0.012	0.017	0.015	0.016	0.021	0.017	0.018	0.017
*N. leucodon*	0.129		0.017	0.012	0.018	0.023	0.018	0.018	0.018
*S. zemni*	0.201	0.194		0.021	0.014	0.022	0.009	0.012	0.013
*N. xanthodon*	0.123	0.100	0.188		0.022	0.029	0.022	0.022	0.021
*S. antiqus*	0.187	0.199	**0.120**	0.196		0.022	0.013	0.014	0.010
*R. synensis*	0.266	0.287	0.286	0.292	0.274		0.022	0.023	0.022
*S. arenarius*	0.200	0.205	**0.055**	0.193	**0.110**	0.282		0.011	0.012
*S. microphthalmus*	0.218	0.211	**0.101**	0.192	**0.131**	0.306	**0.084**		0.014
*S. graecus*	0.196	0.205	**0.112**	0.185	**0.067**	0.290	**0.094**	**0.126**	

Bold—values among five *Spalax* species; underlined—values among three *Nannospalax* superspecies.

## Data Availability

DNA nucleotide sequences were deposited in the NCBI GenBank, and can be assessed at https://www.ncbi.nlm.nih.gov/genbank/ (accessed on 30 March 2022).

## Supplementary Materials

The following supporting information can be downloaded at: https://www.mdpi.com/article/10.3390/ani12091097/s1, Table S1. A list of *16S rRNA* sequences imported from GenBank. See Figure 1 for abbreviations of *N. leucodon* chromosomal forms and their sampling localities (letters in parentheses); Table S2. A list of *MT-CYTB* sequences imported from GenBank. See Figure 1 for abbreviations of *N. leucodon* chromosomal forms and their sampling localities (letters in parentheses); Table S3. Primer sequences applied in the analysis; Figure S1. Geographic distribution of sampling localities of all specimens (sequences) analysed in this study. 1—Zlatibor (RS), 2—Vlasina (RS), 3—Jadovnik (RS), 4—Mačvanski Pričinović (RS), 5—Gvozd Mt. (MN), 6—Beograd (RS), 7—Šumarak (RS), 8—Pančevo (RS), 9—Kajtasovo (RS), Vršački Breg (RS), 11—Stražilovo (RS), 12—Sremski Karlovci (RS), 13—Šuica (BA), 14—Jakupica (MK), 15—Ohrid (MK), 16—Strumica (MK), 17—Lefkothea (GR), 18—Novo Selo (BG), 19—Levadia (GR), 20—Ristovac (RS), 21—Klinovac (RS), 22—Štip (MK), 23—Çorlu (TR), 24—Popova Šapka (MK), 25—Mezőtúr (HU), 26—Józsa (HU), 27—Subotička Peščara (RS), 28—Lipnița (RO), 29—Kars Arpaçay (TR), 30—Karaman (TR), 31—Sarikamiş (TR), 32—Konya (TR), 33—Ankara (TR), 34—Beysehir (TR), 35—Denizili (TR), 36—Malatya (TR), 37—Bingol (TR), 38—Alma (IL), 39—KBZ (IL), 40—Mt. Hermon (IL), 41—El Al (IL), 42—Quenetra (IL), 43—Anza (IL), 44—Lahav (IL), 45—Muhraka (IL), 46—Carmel Mt (IL), 47—Iaşi (RO), 48—Kherson (UA), 49—Novomoszkovsz (UA), 50—Čemerno (BA), 51—Jahorina (BA), 52—Deliblato (RS), 53—Grebenac (RS), 54—Hajdúbagos (HU), 55—Debrecen (HU), 56—Hajdúhadház (HU), 57—Apahida (RO), 58- Kelebia (RS), 59—Čortanovci (RS), 60—Galičica (MK), 61—Pelister Mt. (MK), 62—Bistra Mt. (MK), 63—Eceabat, Canakkale (TR), 64—Kırklareli (TR), 65—Odesa (UA), 66—Balıkesir (TR), 67—Kastamonu (TR), 68—Karabük (TR), 69—Kütahya (TR), 70—Aydın (TR), 71—Isparta (TR), 72—Gökçeada (TU), 73—Judean Mt. (IL), 74—Golan Heights (IL), 75—Galilee Mt. (IL), 76—Krivij Rig (UA), 77—Sânduleşti (RO), 78—Aiton (RO), 79—Dealul lui Dumnezeu (RO), 80—David Valley (RO). *N. l. montanoserbicus*—1,2,3,4; *N. l. hercegoviensis*—5,50,51; *N. l. syrmiensis*—6; *N. l. hungaricus*—7,8,9,10,25,52,53; *N. l. transsylvanicus*—26,54,55,56,57; *N. l. montanosyrmiensis*—11,12,27,58,59; *N. l. monticola*—13; *N. l. makedonicus*—14,15,17,60,61,62; *N. l. strumiciensis*—16; *N. l. epiroticus*—17; *N. l. thracius*—18; *N. l. hellenicus*—19; *N. l. serbicus*—20,21; *N. l. ovchepolensis*—22; *N. l. turcicus*—23,63,64; *N. l. srebarnensis*—28; *N. l. leucodon*—65; *N. l.* unknown—24; *N. xanthodon*—29,30,31,32,33,34,35, 66,67,68,69,70,71,72; *N. xantodon vasvarii*—36; *N. xantodon tuncelicus*—37; *N. ehrenbergi galili*—38,39,75; *N. ehrenbergi golani*—40,41,42,74; *N. ehrenbergi judaei*—43,44,73; *N. ehrenbergi carmeli*—45,46; *S. graecus*—47,79,80; *S. arenarius*—48; *S. microphthalmus*—49; *S. zemni*—76; *S. antiquus*—77,78.
