# Supplementary material for: Cryptic Diversity of the European Blind Mole Rat Nannospalax leucodon Species Complex: Implications for Conservation
Source: Animals (Basel). 2022 Apr 23;12(9):1097. doi: 10.3390/ani12091097 (PMC9105853; doi:10.3390/ani12091097)
Supplement: Supplementary file 1 [file animals-12-01097-s001.zip › animals-1631526-supplementary.pdf]

Table S1. A list of *16S rRNA* sequences imported from GenBank. See Figure 1 for abbreviations of *N. leucodon* chromosomal forms and their sampling localities (letters in parentheses).

GenBank ID	Sampling Locality *	Species/Chromosomal Form	2n/NF	Reference
HQ652170	Mezőtúr, HU	*N. leucodon/ hun* (d)	48/84	Hadid et al. 2012
HQ652165	Šušara, RS	*N. leucodon/ hun* (d)	48/84	Hadid et al. 2012
HQ652155	Józsa, HU	*N. leucodon/ tra* (e)	50/84	Hadid et al. 2012
HQ652173	isolate 915	*N. leucodon/ tra* (e)	50/84	Hadid et al., 2012
HQ652171	Subotička Peščara, RS	*N. leucodon/ msy* (f)	54/86	Hadid et al. 2012
HQ652168	isolate 6063	*N. leucodon/ msy* (f)	54	Hadid, Y. 2010
HQ652174	Lipnița, RO	*N. leucodon/ sre* (s)	48/78	Hadid et al. 2012
HQ652152	Kars Arpaçay, TR	*N. xanthodon*	50	Hadid et al. 2012
HQ652184	Karaman, TR	*N. xanthodon*	60	Hadid et al. 2012
HQ652189	Sarikamiş, TR	*N. xanthodon*	50	Hadid et al. 2012
HQ652185	Konya, TR	*N. xanthodon*	62	Hadid et al. 2012
HQ652176	Ankara, TR	*N. xanthodon*	62	Hadid et al. 2012
HQ652180	Beysehir, TR	*N. xanthodon*	40	Hadid et al. 2012
HQ652181	Denizili, TR	*N. xanthodon*	60	Hadid et al. 2012
HQ652187	Malatya, TR	*N. xantodon/ vasvarii*	60	Hadid et al. 2012
HQ652179	Bingol, TR	*N. xantodon/ tuncelicus*	54	Hadid et al. 2012
HQ652154	Alma, IL	*N. ehrenbergi/ galili*	52	Hadid et al. 2012
HQ652153	Alma, IL	*N. ehrenbergi/ galili*	52	Hadid et al. 2012
HQ652160	KBZ, IL	*N. ehrenbergi/ galili*	52	Hadid et al. 2012
HQ652161	Alma, IL	*N. ehrenbergi/ galili*	52	Hadid et al. 2012
HQ652167	KBZ, IL	*N. ehrenbergi/ galili*	52	Hadid et al. 2012
HQ652164	Mt. Hermon, IL	*N. ehrenbergi/ golani*	54	Hadid et al. 2012

HQ652166	El Al, IL	*N. ehrenbergi/ golani*	54	Hadid et al. 2012
HQ652169	Quenetra, IL	*N. ehrenbergi/ golani*	54	Hadid et al. 2012
HQ652177	Anza, IL	*N. ehrenbergi/ judaei*	60	Hadid et al. 2012
HQ652186	Lahav, IL	*N. ehrenbergi/ judaei*	60	Hadid et al. 2012
JN571137	Muhraka, IL	*N. ehrenbergi/ carmeli*	58	Hadid et al. 2012
NC020756	Mt Carmel, IL	*N. ehrenbergi/ carmeli*	58	Hadid et al. 2012
HQ652192	Iaşi, RO	*S. graecus graecus*	62	Hadid, Y. 2010
HQ652193	Kherson, UA	*S. arenarius*	62	Hadid, Y. 2010
HQ652172	Novomoszkovsz, UA	*S. microphthalmus*	60	Hadid, Y. 2010
KM434232	-	*R. sinensis*		Xu et al. 2014
NC026124	-	*R. sinensis*		Xu et al. 2014

* ISO country code

Table S2: A list of *MT-CYTB* sequences imported from GenBank. See Figure 1 for abbreviations of *N. leucodon* chromosomal forms and their sampling localities (letters in parentheses).

GenBank ID	Sampling Locality*	Species/Chromosomal Form	2n/NF	Reference
JX451841	Čemerno, BA	*N. leucodon/ her* (b)	54/90	Kryštufek et al. 2012
JX451833	Jahorina Mt. BA	*N. leucodon/ her* (b)	54/90	Kryštufek et al. 2012
MN497966	Deliblato, RS	*N. leucodon/ hun* (d)	48/84	Nemeth et al. 2020
JX451843	Grebenac, RS	*N. leucodon/ hun* (d)	48/84	Kryštufek et al. 2012
JX451842	Grebenac, RS	*N. leucodon/ hun* (d)	48/84	Kryštufek et al. 2012
JN656385	Hajdúbagos, HU	*N. leucodon/ tra* (e)	50/84	Nemeth et al. 2013
MN497974	Debrecen, HU	*N. leucodon/ tra* (e)	50/84	Nemeth et al. 2020
MN497973	Hajdúhadház, HU	*N. leucodon/ tra* (e)	50/84	Nemeth et al. 2020
MN497972	Apahida, RO	*N. leucodon/ tra* (e)	50/84	Nemeth et al. 2020
MN497971	Józsa, HU	*N. leucodon/ tra* (e)	50/84	Nemeth et al. 2020

JN656390	Kelebia, RS	*N. leucodon/ msy* (f)	54/86	Nemeth et al. 2013
JN656389	Stražilovo, RS	*N. leucodon/ msy* (f)	54/86	Nemeth et al. 2013
JN656386	Čortanovci, RS	*N. leucodon/ msy* (f)	54/86	Nemeth et al. 2013
JX451840	Galičica, MK	*N. leucodon/ mak* (h)	52/86	Kryštufek et al. 2012
JX451839	Galičica, MK	*N. leucodon/ mak* (h)	52/86	Kryštufek et al. 2012
JX451838	Pelister Mt. MK	*N. leucodon/ mak* (h)	52/86	Kryštufek et al. 2012
JX451837	Pelister Mt. MK	*N. leucodon/ mak* (h)	52/86	Kryštufek et al. 2012
JX451836	Pelister Mt. MK	*N. leucodon/ mak* (h)	52/86	Kryštufek et al. 2012
JX451835	Bistra Mt. MK	*N. leucodon/ mak* (h)	52/86	Kryštufek et al. 2012
JX451834	Bistra Mt. MK	*N. leucodon/ mak* (h)	52/86	Kryštufek et al. 2012
MH300079	Eceabat, Canakkale, TR	*N. leucodon/ tur* (r)	56/76-78	Matur et al. 2018
FJ656300	Kırklareli, TR	*N. leucodon/ tur* (r)	56/76-78	Sözen et al. 2016
MH300078	Eceabat, Canakkale, TR	*N. leucodon/ tur* (r)	56/76-78	Matur et al. 2018
FJ656301	Kırklareli, TR	*N. leucodon/ tur* (r)	56/76-78	Sözen et al. 2016
FJ656299	Kırklareli, TR	*N. leucodon/ tur* (r)	56/76-78	Sözen et al. 2016
JN656388	Odesa, UA	*N. leucodon/ leu* (t)	56/84	Nemeth et al. 2013
FJ656285	Balıkesir, TR	*N. xanthodon*	2n38	Sözen et al. 2016
FJ656298	Kastamonu, TR	*N. xanthodon*	2n60	Sözen et al. 2016
FJ656297	Karabük, TR	*N. xanthodon*	2n60	Sözen et al. 2016
FJ656296	Kütahya, TR	*N. xanthodon*	2n60	Sözen et al. 2016
FJ656295	Kütahya, TR	*N. xanthodon*	2n60	Sözen et al. 2016
FJ656280	Aydın, TR	*N. xanthodon*	2n36	Sözen et al. 2016
FJ656279	Aydın, TR	*N. xanthodon*	2n36	Sözen et al. 2016
FJ656294	Isparta, TR	*N. xanthodon*	2n56	Sözen et al. 2016
FJ656292	Isparta, TR	*N. xanthodon*	2n56	Sözen et al. 2016

FJ656264	Isparta, TR	*N. xanthodon*	2n40	Sözen et al. 2016
FJ656263	Gökçeada, TR	*N. xanthodon*	2n38	Sözen et al. 2016
NC020756	Carmel Mt, IL	*N. carmeli*	58	Hadid et al. 2012
JN571137	Carmel Mt, IL	*N. carmeli*	58	Hadid et al. 2012
JN571136	Judean Mt, IL	*N. judaei*	60	Hadid et al. 2012
JN571135	Judean Mt, IL	*N. judaei*	60	Hadid et al. 2012
JN571134	Golan Heights, IL	*N. golani*	54	Hadid et al. 2012
NC020757	Golan Heights, IL	*N. golani*	54	Hadid et al. 2012
JN571130	Galilee Mt, IL	*N. galili*	52	Hadid et al. 2012
JN571129	Galilee Mt, IL	*N. galili*	52	Hadid et al. 2012
KF021261	Krivij Rig, UA	*S. zemni*	62	Nemeth et al. 2013
KF021259	Novomoszkovsz, UA	*S. microphthalmus*	60	Nemeth et al. 2013
KF021258	Novomoszkovsz, UA	*S. microphthalmus*	60	Nemeth et al. 2013
KF021262	Kherson, UA	*S. arenarius*	62	Nemeth et al. 2013
KF021257	Sânduleşti, RO	*S. antiquus*	62	Nemeth et al. 2013
KF021256	Aiton, RO	*S. antiquus*	62	Nemeth et al. 2013
KF021253	Dealul lui Dumnezeu, RO	*S. graecus*	62	Nemeth et al. 2013
KF021252	David Valley, RO	*S. graecus*	62	Nemeth et al. 2013
M434232	/	*R. sinensis*	/	Xu et al. 2014
C026124	/	*R. sinensis*	/	Xu et al. 2014

* ISO country code

Table S3: Primer sequences applied in the analysis.

**Primer Name**	**Primer Sequence (5′–3′)**	**Reference**

1CB-IF	ATATTTACATGCCAACGGAG	Newly designed
1CB-IR	ATCATTCAGGTTTGATGTGT	Newly designed
1CB-F	TCAACATGATGAAACTTCGG	Modified from [48]
1CB-R	ACTGGCTGCCCGCCGATTCA	Modified from [48]
CYB1-F	TGACATGAAAAATCATCGTTG	Modified from [48]
CYB2-R	TCAAAATGATATTTGTCCTCA	Newly designed
CYB1-NF	GTAAATCACACCCCCTAATCA	Modified from [49]
CYB2-NR	GAACATATCCTATAAATGCG	Modified from [49]
CYB 2-F	AGCATCTCAACATGATGAAA	Modified from [49]
CYB 1-R	CATTTCTGGTTTACAAGAC	Modified from [49]
16 Sar	CGCCTGTTTATCAAAAACAT	Universal
16 SBR OiR	CCGGTCTGAACTCAGATCACGT	Universal
16S-A In-F	TGACTGTGCAAAGGTAGC	Universal

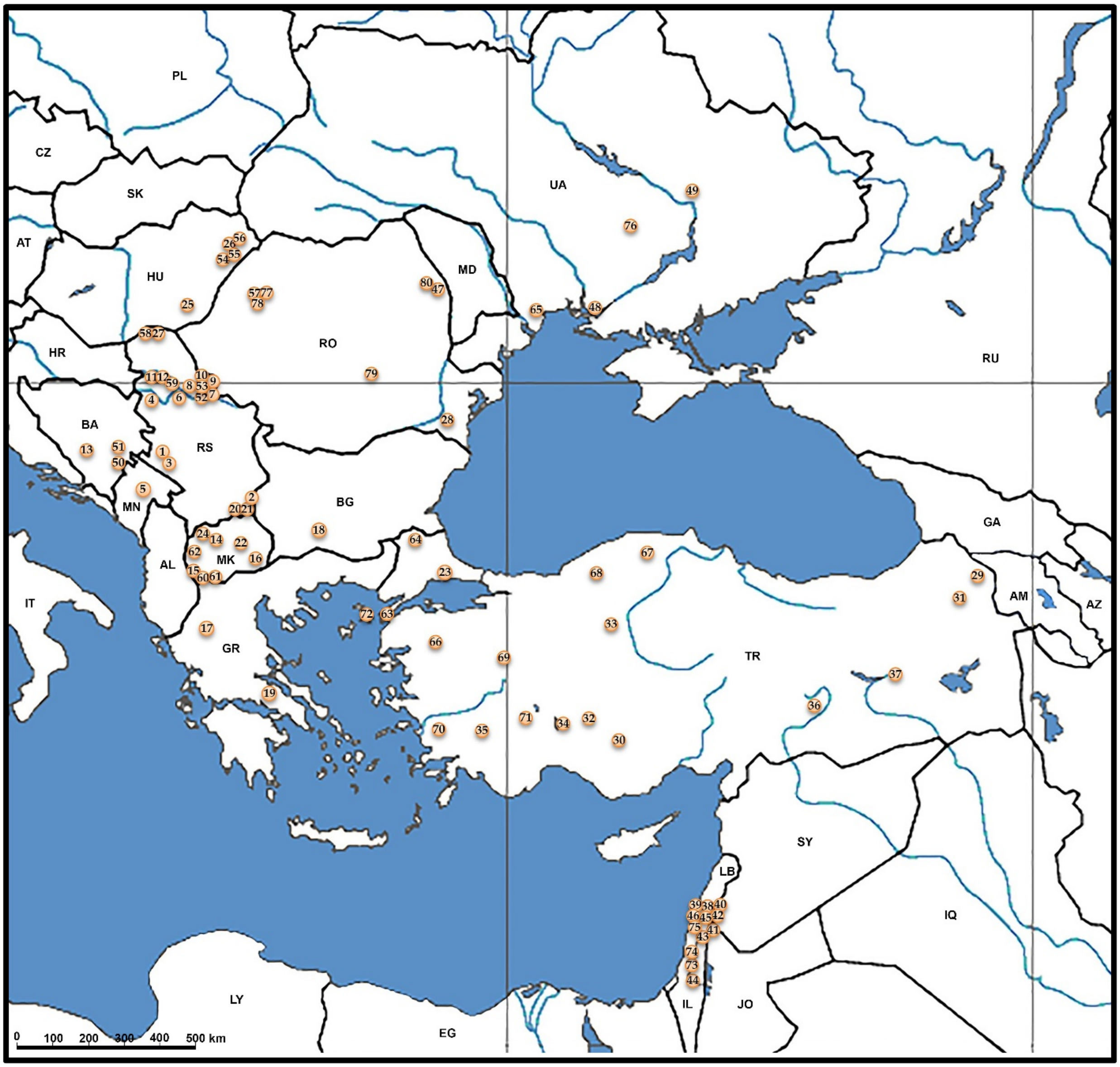

Figure S1. Geographic distribution of sampling localities of all specimens (sequences) analysed in this study.
